# Anti-GD2 Antibody Dinutuximab Beta and Low-Dose Interleukin 2 After Haploidentical Stem-Cell Transplantation in Patients With Relapsed Neuroblastoma: A Multicenter, Phase I/II Trial

**DOI:** 10.1200/JCO.22.01630

**Published:** 2023-02-28

**Authors:** Tim Flaadt, Ruth L. Ladenstein, Martin Ebinger, Holger N. Lode, Helga Björk Arnardóttir, Ulrike Poetschger, Wolfgang Schwinger, Roland Meisel, Friedhelm R. Schuster, Michaela Döring, Peter F. Ambros, Manon Queudeville, Jörg Fuchs, Steven W. Warmann, Jürgen Schäfer, Christian Seitz, Patrick Schlegel, Ines B. Brecht, Ursula Holzer, Tobias Feuchtinger, Thorsten Simon, Johannes H. Schulte, Angelika Eggert, Heiko-Manuel Teltschik, Toni Illhardt, Rupert Handgretinger, Peter Lang

**Affiliations:** ^1^ Department of Hematology and Oncology, University Children's Hospital, Eberhard Karls University Tuebingen, Tuebingen, Germany; ^2^ St Anna Children's Hospital and Children's Cancer Research Institute, Department of Studies and Statistics for Integrated Research and Projects, Medical University of Vienna, Vienna, Austria; ^3^ Department of Pediatrics, Medical University of Vienna, Vienna, Austria; ^4^ Department of Pediatric Hematology and Oncology, University Medicine Greifswald, Greifswald, Germany; ^5^ Department for Studies and Statistics and Integrated Research, Children's Cancer Research Institute, Vienna, Austria; ^6^ Division of Pediatric Hematology-Oncology, Department of Pediatrics and Adolescent Medicine, Medical University Graz, Graz, Austria; ^7^ Division of Pediatric Stem Cell Therapy, Department of Pediatric Oncology, Hematology and Clinical Immunology, Medical Faculty, Heinrich Heine University, Duesseldorf, Germany; ^8^ CCRI, Children's Cancer Research Institute, Vienna, Department of Tumor Biology and Department of Pediatrics, Medical University of Vienna, Vienna, Austria; ^9^ Department of Pediatric Surgery and Pediatric Urology, University Children's Hospital, Eberhard Karls University Tuebingen, Tuebingen, Germany; ^10^ Department for Diagnostic and Interventional Radiology, University Hospital, Eberhard Karls University Tuebingen, Tuebingen, Germany; ^11^ Cluster of Excellence iFIT (Exc 2180) “Image-guided and Functionally Instructed Tumor Therapies,” University of Tuebingen, Germany; ^12^ Children's Medical Research Institute, The Cancer Centre for Children, The Children's Hospital Westmead, University of Sydney, Sydney, Australia; ^13^ Department of Pediatric Hematology, Oncology and Stem Cell Transplantation, Dr von Hauner Children's Hospital, University Hospital, Ludwig Maximilians University Munich, Munich, Germany; ^14^ Department of Pediatric Oncology and Hematology, University Hospital, University of Cologne, Cologne, Germany; ^15^ Department of Pediatric Oncology/Hematology, Charité-Universitaetsmedizin Berlin, Berlin, Germany; ^16^ Department of Hematology and Oncology, Children's Hospital Stuttgart—Olgahospital, Stuttgart, Germany

## Abstract

Patients with relapsed high-risk neuroblastoma (rHR-NB) have a poor prognosis. We hypothesized that graft-versus-neuroblastoma effects could be elicited by transplantation of haploidentical stem cells (haplo-SCT) exploiting cytotoxic functions of natural killer cells and their activation by the anti-GD2 antibody dinutuximab beta (DB). This phase I/II trial assessed safety, feasibility, and outcomes of immunotherapy with DB plus subcutaneous interleukin-2 (scIL2) after haplo-SCT in patients with rHR-NB.

**METHODS:**

Patients age 1-21 years underwent T-/B-cell–depleted haplo-SCT followed by DB and scIL2. The primary end point ‘success of treatment’ encompassed patients receiving six cycles, being alive 180 days after end of trial treatment without progressive disease, unacceptable toxicity, acute graft-versus-host-disease (GvHD) ≥grade 3, or extensive chronic GvHD.

**RESULTS:**

Seventy patients were screened, and 68 were eligible for immunotherapy. Median number of DB cycles was 6 (range, 1-9). Median number of scIL2 cycles was 3 (1−6). The primary end point was met by 37 patients (54.4%). Median observation time was 7.8 years. Five-year event-free survival (EFS) and overall survival from start of trial treatment were 43% (95% CI, 31 to 55) and 53% (95% CI, 41 to 65), respectively. Five-year EFS among patients in complete remission (CR; 52%; 95% CI, 31 to 69) or partial remission (44%; 95% CI, 27 to 60) before immunotherapy were significantly better compared with patients with nonresponse/mixed response/progressive disease (13%; 95% CI, 1 to 42; *P* = .026). Overall response rate in 43 patients with evidence of disease after haplo-SCT was 51% (22 patients), with 15 achieving CR (35%). Two patients developed GvHD grade 2 and 3 each. No unexpected adverse events occurred.

**CONCLUSION:**

DB therapy after haplo-SCT in patients with rHR-NB is feasible, with low risk of inducing GvHD, and results in long-term remissions likely attributable to increased antineuroblastoma activity by donor-derived effector cells.

## INTRODUCTION

Patients with high-risk neuroblastoma (HR-NB) have 5-year survival rates of approximately 50%, whereas patients with metastatic disease at relapse show a 4-year progression-free survival of 6% and overall survival (OS) of 15%.^1,2^ More recently, the combination of anti-GD2 immunotherapy and chemotherapy (chemoimmunotherapy) showed promising overall response rates (ORRs) in patients with relapsed HR-NB (rHR-NB).^3,4^

**CONTEXT**

**Key Objective**
Survival rates for patients with relapsed high-risk neuroblastoma are poor. This study examined feasibility, safety, and response to an immunotherapeutic regimen of dinutuximab beta and low-dose subcutaneous interleukin-2 after haploidentical stem-cell transplantation (haplo-SCT) in patients with relapsed high-risk neuroblastoma.
**Knowledge Generated**
Five-year event-free survival and overall survival from start of trial treatment were 43% and 53%, respectively. Overall response rate and complete response rate in 43 patients with evidence of disease after haplo-SCT were 51% and 35%, respectively. Toxicity profile and treatment-related mortality of the combinational treatment were favorable with a low frequency of graft-versus-host disease.
**Relevance *(S. Bhatia)***
Immunotherapy with dinutuximab beta after haplo-SCT is feasible, safe, and results in long-term remissions. These findings inform the next steps that include definitive randomized trials to determine the role of the individual components of the therapeutic regimens.*
*Relevance section written by *JCO* Associate Editor Smita Bhatia, MD, MPH.

The anti-GD2 antibody dinutuximab beta (ch14.18/CHO; DB) is approved as frontline postconsolidation therapy in HR-NB.^5,6^ DB acts through antibody-dependent cell-mediated cytotoxicity (ADCC) and complement-dependent cytotoxicity (CDC).^7,8^ Previous cytotoxic therapies may impair the ability of natural killer (NK) cells to mediate ADCC.^9^ Therefore, reconstitution of functional NK cells by transplantation of stem cells from haploidentical family donors (haplo-SCT) before immunotherapy is an appealing concept, as NK cells have previously been shown to exert graft-versus-leukemia (GvL) effects.^10^ We hypothesized that NK cell–mediated ADCC after haplo-SCT might induce a graft-versus-neuroblastoma effect and investigated the feasibility, safety, and outcomes of treatment with DB plus subcutaneous interleukin-2 (scIL2) after haplo-SCT in patients with rHR-NB.

## METHODS

### Study Design and Treatment Protocol

#### Screening phase.

This study was a prospective single-arm open-label phase I/II trial. Eligibility criteria were (1) age 1-21 years at trial enrollment, (2) relapsed/refractory International Neuroblastoma Staging System stage 4 neuroblastoma or relapsed *MYCN*-amplified stage 2-3 neuroblastoma, and (3) haplo-SCT as part of the relapse treatment.

The trial protocol did not make recommendations on systemic chemotherapy and local treatment before haplo-SCT (Data Supplement [Appendix 1]). At the discretion of the treating centers, ^131^I-meta-iodobenzylguanidine-(^131^I-mIBG) therapy was given before haplo-SCT. The transplantation followed published guidance.^11,12^ In brief, patients received ex vivo T-/B-cell–depleted peripheral stem cells after myeloablative conditioning (Data Supplement [Appendix 1/2]).^13^ Several transplantation-related factors were important during screening for eligibility and were assessed, including remission status, engraftment, and graft-versus-host-disease (GvHD; Data Supplement [Appendix 1/2]).

#### Trial treatment.

**FIG 1.**
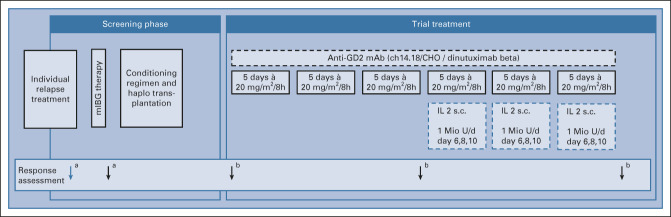
Overview: Screening phase and trial treatment. Response assessment: ^a^Recommended (not part of the trial); ^b^Mandatory: response evaluation before DB treatment, after cycles 3 and 6, after 1 year, and annually thereafter were integral part of this trial. Evaluations included mIBG scintigraphy, bone marrow aspirates (including minimal disease measurement with AIPF), and whole-body MRI or MRI-CT scans of tumor sites. AIPF, automatic immunofluorescence detection system; CT, computed tomography; DB, dinutuximab beta; haplo-SCT, haploidentical stem-cell transplantation; IL2, interleukin 2; mIBG therapy, 131I-meta-iodobenzylguanidine therapy; MRI, magnetic resonance imaging; s.c., subcutaneous.

From day 60 after transplantation, patients without GvHD or acute GvHD (aGVHD) ≤grade 2 were scheduled to receive DB as an 8-hour infusion of 20 mg/m^2^ once per day on five consecutive days, for a total of six cycles given every 4 weeks. To avoid induction of GvHD, low-dose scIL2 was added only in cycles 4-6 on days 6, 8, 10 (1 × 10^6^ IU/m^2^; Fig 1). Patients exhibiting complete response (CR), partial response (PR), or stable disease (SD) after cycle three received three more cycles. In case of response after cycle 6, patients were eligible to receive another three cycles. Following protocol recommendations, continuous morphine infusions were routinely administered during DB treatment.

Immunosuppressive medication had to be stopped before DB treatment. Chemotherapy, experimental anticancer medication, and radiotherapy were not allowed during immunotherapy.

The study Protocol was approved by the appropriate authorities and institutional review boards. All legal guardians and/or patients provided written informed consent before screening. This trial was registered with ClinicalTrials.gov (identifier: NCT02258815) and EudraCT (2009-015936-14).

### Study Assessments

Standard tumor response evaluation was not part of the study protocol but was expected before haplo-SCT, as previous observations suggested remission status as a major factor for outcomes.^13^ Response evaluation using 1993 International Neuroblastoma Response Criteria before DB treatment, after cycles 3, 6, and 9 (if applicable), after 1 year, and annually thereafter was mandatory.^14^ Evaluations included mIBG scintigraphy (International Society of Pediatric Oncology, European Neuroblastoma [SIOPEN] mIBG score^15^), bone marrow aspirates, and whole-body magnetic resonance imaging or magnetic resonance imaging-computed tomography scans of tumor sites, according to the RECIST.^16^ Bone marrow (BM) samples were analyzed according to Mehes et al and later published international guidance, including microscopy and minimal disease (MD) evaluation with automatic immunofluorescence detection of GD2-/CD56-positive neuroblastoma cells.^17-19^ All MIBG scans were submitted to independent central review. For details on response criteria, see appendix 3. Toxicity was recorded according to Common Terminology Criteria for Adverse Events (CTCAE4.0).

### Statistical Analysis

The primary end point ‘success of treatment’ was defined as patients receiving six cycles of DB, alive 180 days after end of trial treatment, without progression, and unacceptable toxicity or acute GvHD ≥ grade 3 or extensive chronic GvHD according to Glucksberg or Seattle classification, respectively.^20,21^

Treatment success of ≥50% was considered relevant with a minimum of 35 evaluable patients for assessing efficacy with a Simon's two-stage design (significance level 5%; power 80%),^22^ followed by a validation group of 25 patients. EFS (events defined as relapse, PD, death, or second malignancy) and OS were estimated using the Kaplan-Meier method, starting from begin of trial treatment, with group comparisons made using the log-rank test. Cumulative incidence (CI) of relapse was estimated accounting for the competing risk of death without relapse/progression and compared using Gray's test. Post hoc univariate and multivariate analyses (MVA; Cox regression) of risk factors were performed.^23^

## RESULTS

### Patient Characteristics and Screening

Seventy patients from four European centers were screened between November 2010 and November 2017; two patients failed screening, and 68 patients were finally enrolled and analyzed. Median age at study entry was 6.5 years (range, 3-20); all but four patients (94.1%) had metastatic disease at relapse. Median observation time from initiation of immunotherapy was 7.8 years.

Most patients (67/68; 98.5%) received chemotherapy for relapse, 39 (57.4%) had surgery, 26 (38.2%) received radiotherapy, and 43 (63.2%) received ^131^I-mIBG therapy before haplo-SCT (Data Supplement [Appendix 1]). Ten patients (14.7%) had anti-GD2 therapy during first-line or relapse treatment. Before haplo-SCT, 16 patients (24%) achieved CR, 39 (59%) demonstrated PR, 11 (17%) showed no response (NR, n = 3), mixed response (MR, n = 4), or PD (n = 4; missing response evaluation in two patients; Table 1). Of note, patients with NR/MR/PD before haplo-SCT had rather limited signs of disease. After an initially good response to individual relapse treatment, these patients demonstrated ≤2 new lesions before transplantation; 50 patients had residual disease before haplo-SCT (median SIOPEN mIBG score 22, range, 0-65). Median times from first relapse to haplo-SCT and trial treatment were 291 days and 415 days, respectively, and 226 days from last relapse to haplo-SCT (Table 1). During screening for eligibility after transplantation, GvHD and engraftment were clinically important factors. Primary engraftment occurred in 65 patients (95.6%). Acute GvHD occurred in 15 patients (22.1%): 13 (19.1%) developed grade 1/2 skin GvHD, 2 (2.9%) developed grade 3 gut GvHD (Data Supplement [Appendix 2b]). Patients without GvHD proceeded to DB treatment after a median time of 91 days (range, 61-363 days). Occurrence of GvHD prolonged time to immunotherapy to a median of 108 days (range, 62-273 days). Median time for all patients was 91 days (range, 61-363 days). No chronic GvHD occurred (Data Supplement [Appendix 2b]).

**TABLE 1.**
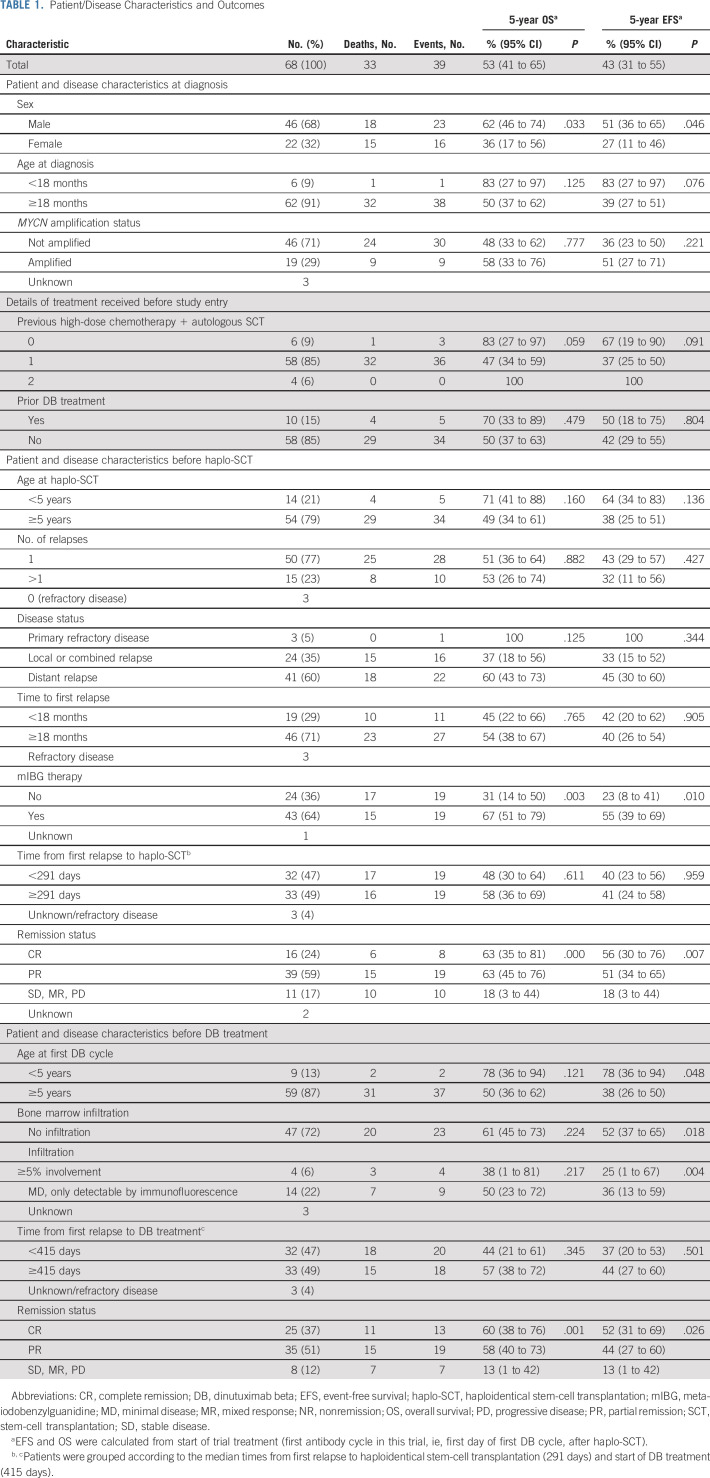
Patient/Disease Characteristics and Outcomes

Most patients improved (48/68; 70.6%) or maintained (18/68; 26.5%) their remission status after haplo-SCT (Table 1).

#### Primary end point.

The primary end point success of treatment was reached by 37 patients (54.4%). Most DB cycles (95.9%) were administered with 20 mg/m^2^/d; prolonged infusion rates (50% decrease/h) were applied in 11.1% of cycles. Lower dosages and prolonged infusion rates followed trial recommendations and were instigated as a result of hypersensitivity reactions. Twenty-nine patients (42.6%) did not complete trial treatment (six cycles): 13 patients (19.1%) because of PD, six because of therapy-related toxicity (hypersensitivity/inflammatory reactions), four because of hemolytic anemia, two because of posterior reversible encephalopathy syndrome (PRES)/central nervous system toxicity, and one each because of human herpes virus 6 (HHV-6) infection and bacterial sepsis; two patients decided to stop immunotherapy. Of the 29 patients discontinuing treatment, 15 received 1-2 cycles and 14 received 3-5 cycles of DB. Twenty-one patients received more than six cycles: three patients received seven cycles, and 18 received nine cycles. In 62 patients (91.2%), scIL2 was administered as prescribed; in five patients (7.3%), administration was unknown. One patient (1.5%) did not receive scIL-2 because of hypersensitivity reactions, but continued with DB treatment.

#### Toxicity.

Treatment-related adverse events (AEs) are summarized in Table 2. Hematologic grade 3/4 AEs occurred in 29 patients (42.6%), with hemolytic anemia reported in six patients (8.8%). Most nonhematologic grade 3/4 AEs were fever, pain, hypersensitivity reactions, capillary leak syndrome, elevated liver enzymes, and central neurotoxicity. Sixty-two patients (91.2%) experienced pain in cycle 1, which decreased to 26 patients (38.2%) by cycle 6. Anaphylactic/inflammatory reactions requiring intensive care treatment were observed in six patients (8.8%). Viral, fungal, and bacterial infections occurred in five patients, three of whom died: one because of HHV-6 infection with encephalitis/pneumonitis and two because of bacterial infections. Severe peripheral neurotoxicity with transient paresthesia occurred in one patient. One patient died after the second DB cycle with signs of encephalitis and/or PRES. This patient had a tumor infiltrating skull and dura, pre-existing absence epilepsy, and Opsoclonus-Myoclonus-Ataxia Syndrome.

**TABLE 2.**
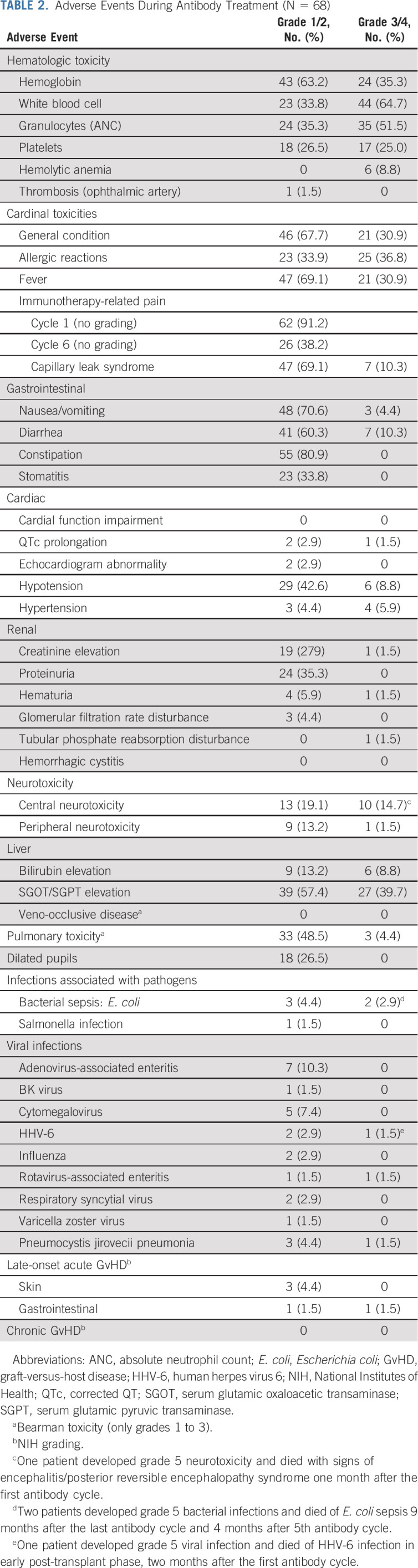
Adverse Events During Antibody Treatment (N = 68)

Occurrence of late-onset aGvHD during DB treatment was low (n = 5; 7.4%). Two patients developed grade 2 and 3 aGvHD of the gut. Three patients (4.4%) developed grade 1/2 skin GvHD, without the need for systemic therapy. No grade 4 GvHD was reported. No additional AEs were observed in the presence of low-dose scIL2.

#### Response assessment.

**FIG 2.**
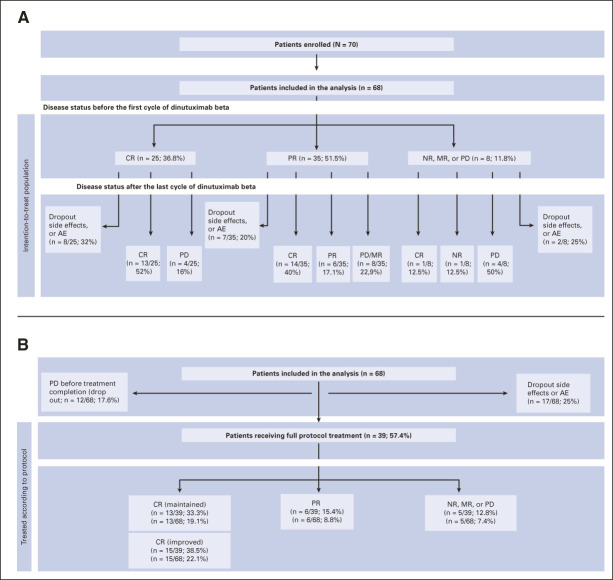
Response to trial treatment: Two patients were ineligible because of screening failure or substantial PD with impaired general condition (Lansky score < 20 before the first antibody cycle). The remaining 68 patients were included in this analysis. (A) The intention-to-treat population (all trial patients), ie, last antibody cycle refers to the last administered cycle of DB received by the respective patient. (B) Patients receiving full protocol treatment (per-protocol population), that is, response represents the remission status after completion of trial treatment, defined as six full cycles of DB treatment. Dropout patients are listed in the upper part of B (PD before treatment completion and dropout side effects or AE). Percentages in the lower part of B refer to patients treated according to protocol (n = 39) and to the whole cohort of 68 patients (intention-to-treat population). CR maintained: patients who started DB treatment in CR; CR improved: patients who achieved CR during/after DB treatment. Event-free survival and overall survival were calculated from start of trial treatment (first antibody cycle in this trial, ie, first day of first DB cycle after haplo-SCT). AE, adverse event; CR, complete remission; DB, dinutuximab beta; EFS, event-free survival; haplo-SCT, haploidentical stem-cell transplantation; MR, mixed response; NR, nonremission; OS, overall survival; PD, progressive disease; PR, partial remission; SD, stable disease.

Before the first DB cycle, 25/68 patients (36.8%) were in CR, 35 (51.5%) were in PR, and eight (11.8%) had NR/MR/PD (Table 1, Fig 2A); 18 patients (26.5%) had measurable disease in the bone marrow, four (5.9%) with infiltration ≥5%, and 14 (20.6%) with MD, only detectable by immunofluorescence. Overall, 13 patients (52%) with CR maintained their CR until the end of trial treatment, and four patients (16%) progressed; 14 patients (40%) with PR achieved CR, while eight (22.9%) had PD. Of the eight patients with NR/MR/PD, four patients progressed during or after treatment, one reached CR (patient had NR with bone metastases; SIOPEN mIBG score 21), and one maintained SD but progressed after the end of treatment (retroperitoneal metastasis; SIOPEN mIBG score not evaluable). In 43 patients with evidence of disease after haplo-SCT, the ORR was 51.2% (n = 22), with a CR rate of 34.9% (n = 15).

A total of 39 patients (57.4%) completed six cycles: 13/68 (19.1%) maintained CR, 15 (22.1%) achieved CR, six (8.8%) had PR, two (2.9%) SD, and three (4.4%) PD at the end of treatment (Fig 2B).

**FIG 3.**
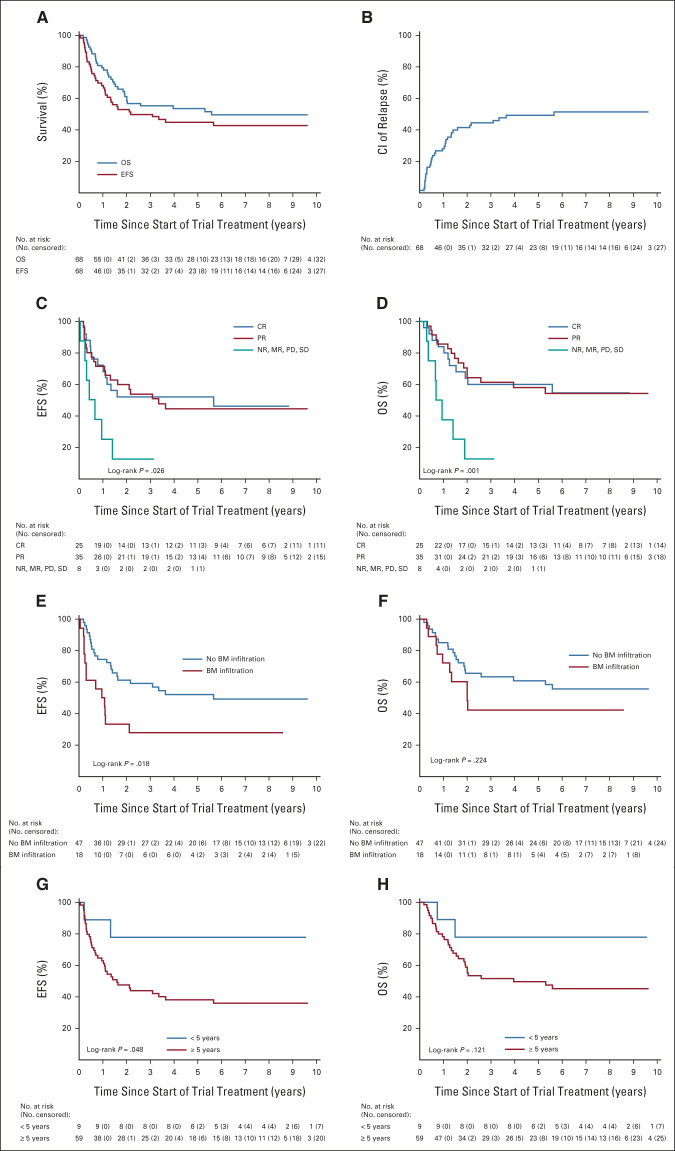
(A) OS and EFS of whole cohort; (B) CI of relapse; (C) EFS: remission status before first DB cycle; (D) OS: remission status before first DB cycle; (E) EFS: BM infiltration before first DB cycle; (F) OS: BM infiltration before first DB cycle; (G) EFS: age at study entry; and (H) OS: age at study entry. Event-free survival and overall survival were calculated from start of trial treatment (first antibody cycle in this trial, ie, first day of first DB cycle after haplo-SCT). BM, bone marrow; CI, cumulative incidence; CR, complete remission; DB, dinutuximab beta; EFS, event-free survival; haplo-SCT, haploidentical stem-cell transplantation; MR, mixed response; NR, nonremission; OS, overall survival; PD, progressive disease; PR, partial remission; SD, stable disease.

At the last follow-up in October 2021, at a median follow-up of 7.8 years, 35/68 (51.5%) patients were alive. The 5-year EFS and OS rates from start of trial treatment of the whole cohort were 53% (95% CI, 41 to 65) and 43% (95% CI, 31 to 55), respectively (Fig 3A). For patients with CR (52%; 95% CI, 31 to 69) or PR (44%; 95% CI, 27 to 60) before immunotherapy, 5-year EFS was better compared with patients with NR, MR, or PD (13%; 95% CI, 1 to 42; *P* = .026). This was also observed for OS (Figs 3C and 3D). The CI of relapse/PD at 5 years was 49% (95% CI, 37 to 61; Fig 3B). Relapse/progression occurred in 34 patients within a median time of 235 days (range, 16-2,067 days) from the first DB cycle. For patients with BM involvement before cycle 1, 5-year EFS was 28% (95% CI, 10 to 49) compared with 52% (95% CI, 37 to 65) for patients with CR in BM (*P* = .018). Similar results were also observed for OS (Figs 3E and 3F). Age ≥ 5 years before DB treatment was associated with a significantly worse EFS but not OS (Figs 3G and 3H).

Causes of death were relapse in 29 patients, infections in three patients, PRES, and secondary malignancy in one patient each. Treatment-related mortality (TRM) at day 100 and after one year was 1.5% [95% CI, 0 to 7] and 7.4% [95% CI, 3 to 15], respectively.

In MVAs, we studied the impact of possible risk factors at relevant time points of the treatment (Table 3). At the time of first diagnosis, we tested significant and/or well-established risk factors, including age, sex, *MYCN* amplification, and time to relapse (Table 1, Data Supplement [Appendix 3]). None of the factors at diagnosis maintained independent prognostic value (Table 3). Factors considered unfavorable before haplo-SCT were BM infiltration, remission status < PR, and no ^131^I-mIBG treatment (Table 1, Data Supplement [Appendix 3]). All three factors kept independent significance for EFS. For OS, no ^131^I-mIBG treatment and poor remission status were significant (Table 3). In MVA of univariate factors before DB treatment, only a remission status < PR maintained independent significance for OS but not for EFS (Table 1 and Table 3).

**TABLE 3.**
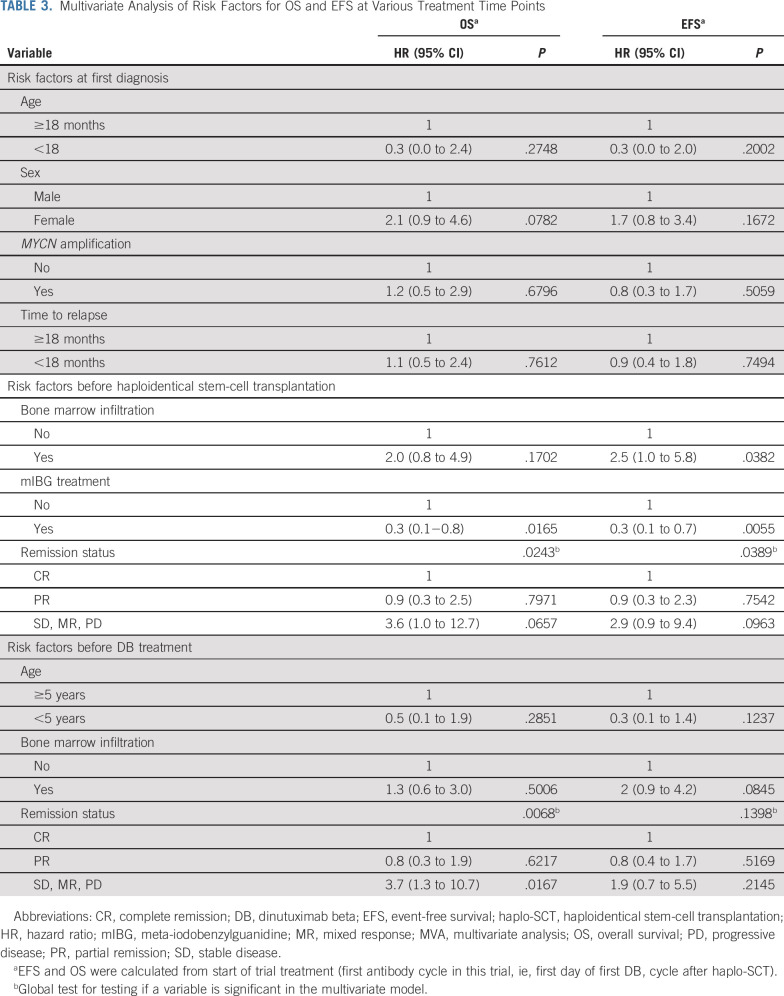
Multivariate Analysis of Risk Factors for OS and EFS at Various Treatment Time Points

## DISCUSSION

Treatment of rHR-NB remains challenging with poor survival rates.^2,24^ Here, we investigated the feasibility, safety, and outcome of DB in combination with low-dose scIL2 after haplo-SCT in a cohort of patients with rHR-NB.

The use of ex vivo T-cell–depleted haplo-SCT takes advantage of high-dose chemotherapy and NK-mediated alloreactive graft-versus-tumor/leukemia GvL effects. In mismatched SCT, NK-mediated GvL effects reduced the relapse rates in patients with leukemia.^10,25,26^ We previously showed that haplo-SCT was associated with low incidence of GvHD and TRM in patients with rHR-NB.^13^ However, the 5-year EFS of 19% indicated insufficient graft-versus-tumor effects.^13^ It has been shown that ADCC can augment post-transplant antitumor activity of donor-derived effector cells.^27,28^ Since DB improves outcomes after autologous SCT during first-line treatment,^5,29^ administration of DB may also augment graft-versus-neuroblastoma effects after haplo-SCT through early expanding and persisting donor-derived NK cells .^30^

Previously, we reported 5-year EFS and OS of 19% and 23%, respectively, in patients with rHR-NB receiving haplo-SCT without antibody treatment, whereas the current trial exhibits 5-year EFS and OS rates of 43% and 53%, respectively.^13^

Disease status before immunotherapy and before haplo-SCT influenced prognosis. Patients with CR or PR before immunotherapy had significantly better 5-year EFS and OS than those with NR/MR/PD. Tumor responses were observed in patients with macroscopic residual disease before DB, as demonstrated by an ORR of 51.2%. Of the 43 patients with disease after haplo-SCT, approximately half reduced their tumor load with DB treatment and 35% achieved CR. This is likely due to an interplay between DB, donor-derived effector cells, and CDC.^30^ Similar results were reported in the HR-NBL1/SIOPEN trial investigating DB after ASCT in frontline therapy^29^; however, it is uncertain whether the autologous immune system can still exert antineuroblastoma activity in the relapse setting after intensive chemotherapy. A limitation of this study is that it only represents the proportion of rHR-HB patients without initial rapid progression during individual relapse treatments. However, on the basis of previously reported relapse trials, our cohort appears to be a comparable collective in terms of risk factors (eg, number of relapses, time to relapse, and *MYCN* amplification status).^2,3,13^ The results of trials evaluating the role of combinational treatment of anti-GD2 antibodies with chemotherapy in rHR-NB have recently reported comparable ORRs.^3,4^ These approaches avoid potential side effects of HSCT, especially GvHD, whereas in our approach, donor-derived effector cells in combination with antibody treatment could provide a stronger, longer-lasting tumor control. Because of the different designs, shorter observation times, and subsequent therapies in several patients in the chemoimmunotherapy trials, a direct comparison with our results is currently limited. A randomized trial would be necessary to demonstrate the superiority of one approach. A combination of both approaches, reinduction with chemoimmunotherapy and consolidation with haplo-SCT followed by DB, could be another option. The majority of our patients were DB-naïve; 10 patients received anti-GD2 therapy during relapse or frontline treatment. OS, EFS, and ORR did not differ significantly between DB-naïve or previously exposed patients. Since repeated anti-GD2 antibody exposure in the relapse setting has been shown to be effective, we assume that this also applies to our approach.^3^

We performed two additional retrospective analyses evaluating factors at diagnosis, at relapse, and those related to haplo-SCT. None of the factors at diagnosis maintained independent prognostic value, likely attributable to the relatively small sample size. One significant factor was ^131^I-mIBG therapy before conditioning given in some patients with residual mIBG-avid disease. Interestingly, independent of remission status before haplo-SCT, ^131^I-mIBG therapy had a significantly positive influence on OS and EFS in univariate and MVA. In vitro data suggest that radiation can induce immunogenic tumor cell death and release of tumor-specific antigens, NK cell ligands, and stress-inducible proteins, which could be identified and attacked by the donor-derived immune system and NK cells cotransfused during haplo-SCT.^31,32^ Remission < PR before haplo-SCT as well as before DB treatment was another factor with independent prognostic value. In univariate analysis, female sex was associated with worse OS, which was not confirmed in MVA.

The toxicity of haplo-SCT was acceptable with low TRM and aGvHD rates (7.5%), which was lower than that reported after allogeneic SCT with matched donors.^33^ Only two patients developed aGvHD grade 2 and 3 each during DB treatment. These cases can either be considered as late-onset aGvHD (>100 days post-transplant), or de novo GvHD induced by the antibody treatment. In both patients, DB was continued after resolution of GvHD without recurrence. Hemolytic anemia is a well-known complication after allogeneic SCT with an incidence of about 6%,^34^ we cannot exclude that the antibody treatment might have induced or aggravated the hemolysis seen in 9% of our patients. The most frequently reported grade 3/4 AEs observed here were similar to those reported during DB treatment after ASCT.^29^

Three patients died during DB therapy because of HHV-6 infection, bacterial infection, and PRES. The infections were considered to be associated with intensive pretreatment and SCT. The encephalopathy might have occurred due to a combination of disease-, transplant-, and antibody-related toxicities. Relapse remained the major cause of death with a CI of 49% at 5 years.

For further optimization, we suggest replacing the CD3/CD19 depletion by T-cell receptor αβ/CD19-depleted grafts, which results in accelerated immune reconstitution and allows cotransfusion of additional γδT cells with potential antitumor and antiviral activity.^35^ Toxicity might be reduced by using the 10-day continuous infusion for DB as previously reported.^36^ The use of checkpoint inhibitors to optimize the efficacy of DB is also currently being explored.^37^ The additional use of scIL2 is questionable since our trial was not designed to evaluate specific effects of scIL2.^30^ SIOPEN data showed that adding scIL2 to DB in the frontline setting does not improve efficacy but increases toxicity.^29^ Thus, we believe that the addition of IL-2 should not be considered in future trials.

In summary, DB therapy after haplo-SCT demonstrated antitumor activity with acceptable toxicity in patients with rHR-NB and was associated with notable EFS and OS among patients who had achieved at least PR with previous therapy. Further prospective and randomized trials are warranted to evaluate the contribution of each component of the approach, and larger cohorts are needed to allow better risk stratification and patient selection.

## Data Availability

The anonymized data from this study that underlie the results reported in this article will be made available, beginning 12 months and ending 5 years after this article's publication, to any investigators who sign a data access agreement and provide a methodologically sound proposal to peter.lang@med.uni-tuebingen.de. The trial protocol will also be made available, as will a data fields dictionary.
